# Differences in the Detailed Milk Mineral Composition of Italian Local and Saanen Goat Breeds

**DOI:** 10.3390/ani9070412

**Published:** 2019-07-02

**Authors:** Sarah Currò, Massimo De Marchi, Salvatore Claps, Angela Salzano, Pasquale De Palo, Carmen L. Manuelian, Gianluca Neglia

**Affiliations:** 1 Department of Agronomy, Food, Natural resources, Animals and Environment, University of Padova, Viale dell’Università 16, 35020 Legnaro (PD), Italy; 2 Council for Agricultural Research and Analysis of Agricultural Economy Analysis, Research Centre for Animal Production and Aquaculture, S.S.7 Via Appia, 85051 Bella Muro (PZ), Italy; 3 Department of Veterinary Medicine and Animal Production (DMVPA), University of Naples Federico II, Naples, Via Federico Delpino 1, 80137 Napoli (NA), Italy; 4 Department of Veterinary Medicine, University of Bari Aldo Moro, S.P. per Casamassima km 3, 70010 Valenzano (BA), Italy

**Keywords:** doe, indigenous breed, lactation, major mineral, trace element

## Abstract

**Simple Summary:**

This study quantified major and trace minerals in milk of five Italian local goat breeds and a cosmopolitan goat breed throughout lactation. Significant differences were detected in milk minerals composition across week of lactation, with greater concentration at the end than at the beginning of the lactation for almost all minerals, while only P, Mg and Zn milk content differed among breeds. Due to the growing interest of consumers in goat milk and derived products, the characterisation of milk mineral contents could contribute to valorise autochthonous breeds.

**Abstract:**

Very little information about local breed goat milk is available, which is relevant for biodiversity preservation and local cheese production. This study aimed to evaluate the effect of breed and week of lactation on milk mineral profile of five Italian local breeds (Garganica, Girgentana, Jonica, Maltese and Mediterranean Red) and a cosmopolitan breed (Saanen). Sixty goats (10 per breed) from an experimental farm were enrolled in the study and sampled every 2 weeks for milk gross composition analysis. In addition, an individual milk sample was collected monthly from 42 goats (seven goats per breed) for mineral determination through inductively coupled plasma optical emission spectrometry. Data were analysed using a mixed linear model with repeated measures, including breed and week of lactation as fixed effects. Week of lactation affected mineral contents, except for B, being lower in early than late lactation, whereas, breed affected only P, Mg and Zn. Mediterranean Red and Jonica breeds’ milk was richer in P than Maltese, and in Zn than Maltese, Girgentana and Saanen breeds. For Mg, only Saanen differed from Maltese. Such information might be useful for the valorisation of goat milk produced by autochthonous breeds.

## 1. Introduction

Europe produces approximately 15% (2.8 × 10^6^ tons) of the world’s goat milk (18.6 × 10^6^ tons) [1] and 95% of this amount is transformed into dairy products [2]. Goat cheese production corresponds to 2.3% (52 × 10^4^ tons) of worldwide cheese production (23 × 10^6^ tons) [1], and several goat cheeses have protected designation status. In the last decade, the Italian goat milk and cheese production has increased 17% (24.9 to 29.2 × 10^3^ tons) and 32% (3.4 to 4.5 × 10^3^ tons), respectively [1]. Consumers’ interest in goat milk and derived products is mainly related to its better digestibility for infants, the elderly and patients with gastrointestinal disorders [3,4] compared with cow milk; the smaller fat globule size and lower αs1 casein content of goat compared with cow milk are the major reasons of the difference in digestibility and allergenicity [5,6]. Sanz Ceballos et al. [5] reported greater amounts of Ca, P, Mg and Cu in goat compared with cow milk under identical environmental conditions. In addition, goat milk shows lower lactose content with a greater abundance of oligosaccharides derived from lactose, that positively affect human health for their prebiotic and anti-infective nature [7]. Moreover, goat milk contains more short fatty acids, n3, n6 and vitamin A than cow milk. Furthermore, fermented goat milk has a probiotic component that is maintained quite constant during the whole shelf life of the product for the low pH (6.47) and the buffering effect; in fact, fermented goat milk is considered a means to provide and improve probiotic intake in the human diet [7]. 

Milk minerals play an important role in human health [4,6] and milk coagulation ability [8]. In particular, Ca and P affect bone and teeth structure and muscular function, and Zn influences skin health and carbohydrate metabolism [6]. Copper and Fe are involved in the haemoglobin synthesis and transportation [9]. Moreover, Barrionuevo et al. [9] reported that goat milk consumption favoured Fe and Cu absorption in healthy rats and in those with malabsorption syndrome compared with cow milk. From a technological point of view, Ca and P strongly affect the milk coagulation ability and the firmness of the coagulum at the end of the coagulation process [8].

According to the literature, breed and stage of lactation affect mineral content of goat milk [10,11]. However, studies are mainly focused on cosmopolitan breeds such as Saanen, Toggenburg, Alpine and Anglo-Nubian [12,13], whereas local breeds are often neglected. In the last decades, local breeds have been progressively replaced with cosmopolitan breeds with the aim of increasing milk production. Nevertheless, studies on local breeds have revealed that milk gross composition is generally better in local than cosmopolitan breeds [12]. Moreover, the replacement of native with high productive breeds is responsible for the reduction of the variability within species, the loss of niche products related to specific breeds and the loss of historical heritage of the country [14]. The FAO database [15] reported that among the 55 Italian goat breeds, 61% are endangered and 29% are in unknown conditions of risk.

Nowadays, the assessment of milk quality is not only based on traditional components (i.e., fat and protein contents), but also on more specific compounds such as protein profile, fatty acid profile, and mineral content. However, information on those compounds and differences among breeds is very scarce. Due to the growing interest of consumers towards goat milk and dairy products, mineral and fatty acid composition of milk from local goat breeds might be useful to valorise their productions. Because some minerals are correlated with protein and fat content in milk [16], and differences in fat and protein content have been reported between Saanen and local breeds [12], we expect that breeds present differences in milk mineral content, in particular when local are compared with cosmopolitan breeds. Therefore, the aim of the present study was to characterise milk mineral contents of 5 local Italian goat breeds and to compare them with the cosmopolitan Saanen breed.

## 2. Materials and Methods 

### 2.1. Animals and Management Conditions

This research was conducted from February to August 2016 in the experimental farm of the Council for Agricultural Research and Analysis of Agricultural Economy Analysis, Research Unit of Extensive Animal Production (CRA-ZOE, Potenza, Italy). Experimental procedures and animal care conditions followed the recommendations of European Union directive 86/609/EEC. All animals were in the same farm under the same management conditions. Breeds included in the study were Garganica (GA), Girgentana (GI), Jonica (JO), Maltese (MA), Mediterranean Red (MR) and Saanen (SA). A general description of the 6 breeds is reported in Currò et al. [17].

A total of 60 does (10 per breed) that kidded twins in February 2016 were enrolled in the study. Animals had similar body condition score at parturition (between 2.5 and 3.0; 1 = very thin to 5 = very fat, with 0.5 point-increment [18]), were from parity 1 to 5 (balanced among breeds) and their BW at the beginning of lactation averaged 48 ± 4 kg for GA, 42 ± 6 kg for GI, 47 ± 6 kg for JO, 46 ± 5 kg for MA, 48 ± 3 kg for MR and 64 ± 7 kg for SA. Kids were kept with their dams until 40 days after birth and temporarily separated 24 h before every sampling day. Does were milked twice a day (morning and evening) in a double 24-stall herringbone low-line milk pipeline milking parlour (Alfa Laval Agri; Monza, Italy) equipped with recording jars and electronic pulsators at a vacuum of 38 kPa, 90 pulses/min and 60% pulsation ratio. The pre-milking phase included only fore stripping without any preparation of udder and teats. None of the does presented mastitis events throughout the trial.

During the study, does grazed together during the day in a natural pasture (8h/day) and were supplemented with polyphite hay ad libitum in the shelter, composed of 60–65% of grasses (mainly *Avena sativa* L.) and 35–40% of legumes (mainly *Vicia sativa* L.) [chemical composition: 89.10% of dry matter (DM), 15.10% of crude protein on DM, 52.60% of neutral detergent fibre on DM, and 1.10 Mcal/kg of net energy of lactation]. In addition, a commercial concentrate was offered to each doe in the milking parlour according to their requirements, considering the mean body weight and mean milk production every 15 days for each breed, following National Research Council recommendations [19]. Saanen received between 0.8 and 1.3 kg/day, and local breeds received between 0.5 and 1.0 kg/day, being the greatest amount at the beginning and the lowest at the end of the lactation. Throughout the study all goats consumed the total amount of concentrated offered, and no spillage was observed. Concentrate included maize, wheat bran and flour, maize and sunflower germ flours, sugar beet molasses, soybean meal (48% crude protein), calcium carbonate, sodium chloride, sodium bicarbonate, I (5 mg/kg), Mn (50 mg/kg) and Zn (125 mg/kg). The chemical composition of the commercial concentrate was 88.20% of DM, 21.70% of crude protein on DM, 23.00% of neutral detergent fibre on DM and 1.77 Mcal/kg of net energy of lactation. 

### 2.2. Sample Collection and Chemical Analysis

Individual milk yield (kg/day) was recorded for each doe as the sum of morning and evening milkings from 2 to 30 weeks of lactation using the recording jars in the milking parlour. A total of 840 individual milk samples (50 mL each) were collected every two weeks and analysed for gross composition. Moreover, every month an additional milk sample from 42 out of the 60 goats (50 mL; n = 252; 7 goats per breed) was collected for mineral contents analysis.

Milk samples used for milk gross composition were stored at 4°C and analysed in the milk laboratory of the Breeders Association of Basilicata region (Potenza, Italy). Fat-corrected milk at 3.5% (FCM3.5%, kg/day) was calculated according to Pulina et al. [20]:

FCM3.5% = milk yield (kg/day) × (0.634 + 0.1046 × fat%).(1)

Fat, protein and lactose percentages were determined using MilkoScan FT6000 (Foss Electric, Hillerød, Denmark). Somatic cell count (SCC, cells/mL) was assessed by Fossomatic FC (Foss Electric,) and transformed to somatic cell score (SCS) through the following formula [21]:

SCS = 3 + log2(SCC/100 000).(2)

Milk samples used for minerals determination were stored at -80°C and analysed in the laboratory of the Department of Agronomy, Food, Natural resources, Animals and Environment of the University of Padova (Legnaro, Italy). Major (K, Ca, P, Na, S and Mg) and trace minerals (Zn, B, Sr, Ba, Fe, Al, As, Cr, Cu and Li) were determined using inductively coupled plasma optical emission spectrometry (ICP-OES), Ciros Vision EOP (Spectro Analytical Instruments GmbH, Kleve, Germany) after mineralisation of the sample with nitric acid in closed vessels by a microwave system (Ethos 1600 Milestone S.r.l., Sorisole, Italy), following the procedures described in Manuelian et al. [22]. Instrument operating parameters (sample aspiration rate of 2 mL/min, plasma power 1350 W, coolant flow 11 L/min, auxiliary flow 0.60 L/min, nebulizer flow 0.75 L/min and integration time of 28 s) were optimized for acid solution. Calibration standards were prepared from single element solutions (Inorganic Ventures, Christiansburg, VA, USA) in a concentration range between 0 and 100 mg/L and matched with 5% HNO_3_ (vol/vol) solution using 65% HNO_3_ Suprapur (100441, Merck, Darmstadt, Germany). The wavelengths used to determine the minerals were: 766.941 nm for K, 317.933 nm for Ca, 178.287 nm for P, 589.592 nm for Na, 182.034 nm for S, 285.213 nm for Mg, 213.856 nm for Zn, 249.677 nm for B, 407.771 for Sr, 455.404 nm for Ba, 259.941 nm for Fe, 167.078 nm for Al, 189.042 nm for As, 267.716 nm for Cr, 324.754 nm for Cu and 670.780 nm for Li. However, Al, As, Cr, Cu and Li were below the limit of detection of the instrument (0.01 µg/kg of DM) and were not further considered in this study. 

### 2.3. Statistical Analysis

The final dataset consisted of 815 records for milk yield, FCM3.5%, SCS and gross milk composition, and 217 records for milk mineral composition. Sources of variation of milk yield, FCM3.5%, SCS, and gross and mineral composition were investigated using the MIXED procedure of SAS v9.4 (SAS Inst. Inc., Cary, NC, USA) with repeated measures, according to the following mixed linear model:

y_ijk_ = µ + Breed_i_ + Week_j_ + (Breed × Week)_ij_ + Goat_k_(Breed_i_) + ε_ijk_, (3)

where y_ijk_ is the dependent variable (milk yield, FCM3.5%, SCS, fat, protein, lactose or each mineral); µ is the overall intercept of the model; Breed_i_ is the fixed effect of the *i*th breed (*i* = GA, GI, JO, MA, MR, SA); Week_j_ is the fixed effect of the *j*th week of lactation (*j* = 1 to 14 for milk yield, FCM3.5%, SCS, fat, protein and lactose, corresponding to every 2-week sampling; *j* = 1 to 6 for each mineral, corresponding to every 4-week sampling); (Breed × Week)_ij_ is the fixed interaction effect between breed and week of lactation; Goat_k_(Breed_i_) is the random effect of the *k*th goat nested within the *i*th breed ~N (0, σ^2^_Goat(Breed)_); and ε_ijk_ is the random residual ~N(0, σ^2^_ε_). In a preliminary analysis, the interactions Parity × Week of lactation and Breed × Parity were not significant and thus they were removed from the final model. Multiple comparisons of least squares means were performed for the main effects of breed and week of lactation using Tukey’s test adjustment. Values are shown as least squares means ± standard error and significance was declared at *p* < 0.05, unless otherwise indicated.

## 3. Results 

### 3.1. Descriptive Statistics

Descriptive statistics for milk yield, FCM3.5%, SCS, fat, protein, lactose, major and trace minerals are reported in Table 1. Milk yield, SCS, fat, protein and lactose averaged 1.18 kg/day, 5.61 units, 3.97%, 3.36% and 4.48%, respectively. As expected, milk yield and FCM3.5% were the most variable traits [coefficient of variation (CV) = 45% and 41%, respectively] followed by SCS (CV = 34%). A lower variation was observed for fat (CV = 27%), protein (CV = 17%) and lactose (CV = 7%).

The most abundant mineral in goat milk was K (1662 mg/kg) followed by Ca (1067 mg/kg) and P (796 mg/kg; Table 1). The other major minerals had an overall concentration between 107 mg/kg (Mg) and 348 mg/kg (Na; Table 1). Among trace minerals, the most abundant was Zn (2.70 µg/g) followed by B (1.42 µg/g). The other trace minerals ranged from an overall mean of 0.31 µg/g (Fe) to 0.81 µg/g (Sr). The CV was lower for major than trace minerals: in particular, the CV of major minerals ranged from 14% (K) to 20% (Na and Mg), and CV of trace minerals from 27% (Zn) to 91% (Ba).

### 3.2. Breed Effect

Breed strongly affected milk yield, FCM3.5%, fat, protein, lactose and SCS (*p* < 0.01; Table 2). Saanen had greater milk yield and SCS but lower fat content compared with the local breeds. However, in terms of FCM3.5% SA had similar milk production than JO and MA. Moreover, milk protein content of SA differed only from MA, and lactose content was similar between the SA and the GA. The order of abundance of major and trace minerals was similar in the different breeds (Table 2). Also, mineral contents slightly differed among breeds, and differences concerned only P (*p* < 0.001), Mg (*p* = 0.048) and Zn (*p* < 0.001). The greatest P content was detected for MR and JO, whose milk had on average 121 mg/kg more P than MA and GI breeds (*p* < 0.001). No differences between SA and the local breeds were observed for P. Regarding Mg content, the only significant difference was observed between SA and MA (+25 mg/kg for SA; *p* < 0.05). Mediterranean Red and JO breeds had the greatest Zn content, producing about 0.90, 0.72 and 0.69 µg/g more Zn than MA, GI and SA breeds, respectively.

### 3.3. Effect of Stage of Lactation

**Figure 1:**
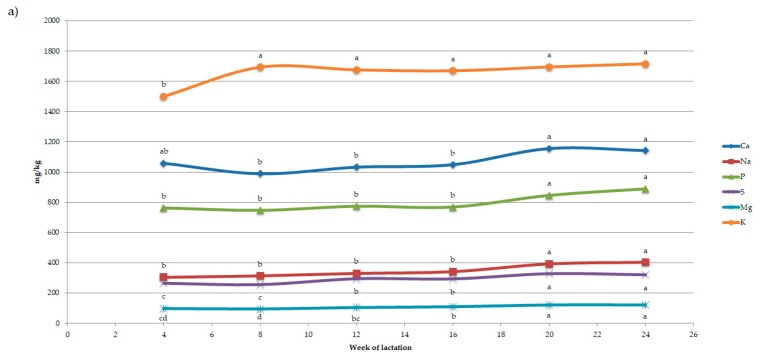

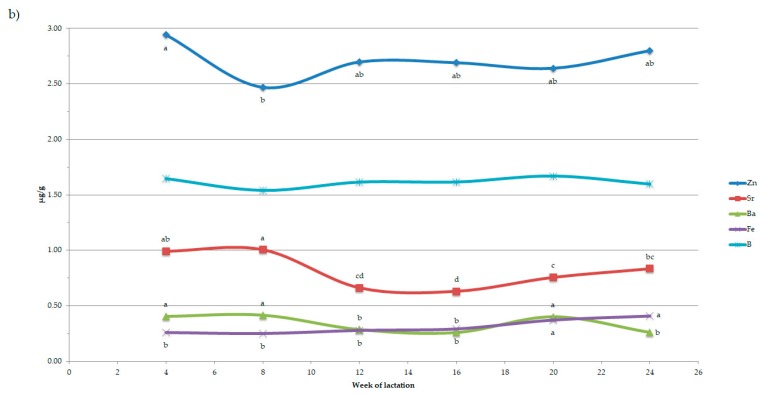
Least squares means of (**a**) major and (**b**) trace minerals of goat milk during lactation. Least squares means with different superscripts within a mineral differ significantly (*p* < 0.05).

Week of lactation affected (*p* < 0.001) milk yield, FCM3.5%, fat, protein, lactose and SCS. Milk yield, FCM3.5% and lactose decreased by 67%, 63% and 11%, respectively, across lactation, whereas SCS, fat and protein increased by 60%, 44% and 37%, respectively. Week of lactation affected also major and trace minerals (*p* < 0.001; *p* = 0.023 for Zn) with the exception of B (*p* = 0.83). The lowest milk contents of K (1500 mg/kg), Ca (989 mg/kg), P (747 mg/kg), Na (304 mg/kg), S (256 mg/kg) and Mg (96 mg/kg) were observed in early lactation (4th and 8th week of lactation), which corresponded to the peak of lactation (Figure 1). In particular, the lowest K amount was detected in the 4th week of lactation (1500 mg/kg), whereas its amount increased in the 8th week of lactation (+12.9%) and remained stable until the end of lactation. Conversely to K trend, the content of Ca, P and Na was quite stable from 4th to 16th week of lactation, incremented +10% for Ca and P and 15% for Na between the 16th and late lactation (20th and 24th week of lactation). Moreover, S and Mg showed the lowest content in early lactation and increased of 15% during mid lactation (12th and 16th week of lactation) reporting the greatest content (Ca, 1156 mg/kg; P, 889 mg/kg; Na, 404 mg/kg; S, 329 mg/kg; Mg, 122 mg/kg) in late lactation (20th and 24th week of lactation). The greatest Zn content was found at the 4th week of lactation (2.94 µg/g), which differed significantly from the 8th week of lactation (2.47 µg/g). Intermediate values of Zn content were observed from the 12th to 24th week of lactation (2.64 to 2.80 µg/g). Strontium showed the greatest content in early lactation (1.00 µg/g from the 4th to 8th week of lactation), decreased by 37% until the 16th week (0.63 µg/g) and increased thereafter again until the 24th week of lactation (0.83 µg/g). 

Barium showed an erratic pattern throughout lactation: the greatest values were obtained in early (4th and 8th week of lactation) and in the 20th week of lactation (0.40 µg/g), whereas the lowest were observed in mid (12th and 16th week of lactation) and in the 24th week of lactation (0.27 µg/g). Iron showed the lowest amount between the 4th and 16th week of lactation and increased in late lactation, where it reached the greatest content (0.39 µg/g).

## 4. Discussion

### 4.1. Means and Variation of Milk Gross Composition and Mineral Content

Overall, means of milk yield and fat, protein and lactose contents were consistent with values of goat milk production and composition reported by Muehlhoff [23]. The average SCS observed in our study (SCS = 5.61) was similar to that reported by Niero et al. [24] for goat milk (SCS = 5.74). The greatest variability of milk yield was expected because the present study dealt with milk samples collected across a complete lactation and included several breeds. The variation observed for FCM3.5% (CV = 41%) was greater than the one reported by Bonanno et al. [25] for GI (CV = 29%) when evaluating the feed effect on 37 goats during 3 months (from April to May). The variability of SCS (CV = 34%) was in agreement with Vacca et al. [26], who reported a similar variation in individual milk samples of six goat breeds (SA, Camosciata delle Alpi, Murciano-Granadina, MA, Sarda and Sarda primitive). Also, the variability observed for fat, protein and lactose contents agreed with findings of Niero et al. [24].

Regarding milk minerals, the greater content of K and Ca compared with other minerals in goat milk was in agreement with Kondyli et al. [3] in local Greek goats and StrzaŁkowska et al. [27] in Polish White improved goats. Nevertheless, Park and Chukwu [11] reported greater content of P (1410 mg/L) than Ca (1389 ml/L) and K (989 mg/L) in milk of French Alpine and Anglo-Nubian goat breeds during the first 5 months of lactation. Milk of local goats of Canary Island [28] had greater Ca (1340 mg/kg), Na (510 mg/kg) and Mg contents (120 mg/kg) and lower K content (1240 mg/kg) compared with the present study. The variability observed for K, P and Ca in our study was similar to the CV reported by StrzaŁkowska et al. [27] in Polish White improved goats. On the other hand, the variability reported by García et al. [28] for Ca (CV = 18%) and K (CV = 16%) was similar to the CV reported in the present study, whereas they observed a greater variability for Mg (CV = 25%).

Regarding trace element, the overall Zn content was lower than Zn contents reported by García et al. [28] (3.20 µg/g), Kondyli et al. [3] (3.80 µg/g) and Güler [29] (4.68 µg/g), and the B content was lower than that reported by Güler [29] (16.9 µg/g), and Şanal et al. [30] (8.09 µg/g) in a Turkish’s local goat breed. The lower contents of B, Sr, Ba and Fe in the present study compared with Güler [29] (16.9, 1.10, 0.99 and 3.88 µg/g, respectively) could be related to the period of lactation considered; indeed, we studied the complete lactation whereas Güler [29] considered milk from late lactation, where milk yield decreases and milk components become more concentrated. 

### 4.2. Breed Effect on Milk Mineral Content

The differences of milk gross composition among breeds have been previously discussed in Currò et al. [17]. Very few studies have assessed the mineral composition of goat milk and the differences among breeds. In fact, milk from Anglo-Nubian and French Alpine goats differed in Na and K content [11]. Trancoso et al. [12] found breed differences for Ca, P, Mg, Na, K and Fe among several Portuguese breeds (Serrana, Serpentina, Charnequeira and Algarvia) and SA reared in different regions. Moreover, those authors observed significant differences between the two different ecotypes (Trasmontana and Ribatejana) of Serrana breed, which suggested that those differences were likely related to the feeding. On the other hand, Mestawet et al. [13] did not observe significant variation for Ca, P, K, Mg, Na, Zn and Fe content among four Ethiopian goat genotypes (Somali, Arsi-Bale, Boer and Toggenburg×Arsi-bale crossbred). A greater content of P and Mg in milk (being part of the casein micelles) could indicate better milk coagulation capacity [31]. However, to compare milk minerals content among studies, differences in botanical species of grazing area, feeding strategies and drinking water composition [32], and the different analytical methods used in each study should be considered because they have an impact on milk mineral contents [33].

### 4.3. Effect of Stage of Lactation on Milk Mineral Content

The differences of milk gross composition among week of lactation have been previously discussed in Currò et al. [17]. The effect of week of lactation on mineral profile in goat milk has been reported by other authors. Nevertheless, the pattern described in the present study for K was opposite compared with findings of Park and Chukwu [11]. Those authors observed the greatest K amount at the 4th week of lactation for Anglo-Nubian goats (1322 mg/L) and at the 8th week of lactation for French Alpine goats (1505 mg/L), with decreasing values thereafter until the 21st week of lactation (530 mg/L at and 675 mg/L, respectively). A possible reason to explain differences in the described pattern for K content between our study and Park and Chukwu [11] could be the *ad libitum* access to a ration rich in K (1.82% of DM) until the 12th week of lactation in the latter study. On the other hand, the pattern observed for Ca, P, Na, S and Mg in the present study agreed with results described in Ethiopian goat breeds by Mestawet et al. [13]. Antunović et al. [34] and Park and Chukwu [11] detected the greatest Ca content at the end of the lactation in Croatian (1589 mg/kg) and Anglo-Nubian (1538 mg/kg) goat breeds, respectivily. 

Zinc trend reported in our study was in agreement with Antunović et al. [34] who reported the lowest Zn content (2.40 µg/g) at the 8th week of lactation and an increment thereafter until the 21st week of lactation (3.60 µg/g) in Croatian goats. However, a different trend for Zn was reported by Kondyli et al. [3] in Greek local goats with a grazing period from April to July. Those authors observed the greatest Zn content at the 6th week of lactation (4.6 µg/g) and the lowest at the end of lactation (3.1 µg/g). Contrary to the pattern of Fe described in the present study, Kondyli et al. [3] and Antunović et al. [34] reported a stable Fe content in milk through the whole lactation in Greek and Croatian goats, respectively. In addition, StrzaŁkowska et al. [27] observed in White Polish goats an erratic trend of milk Fe content through lactation with maximum values in the 1st, 7th and 10th weeks of lactation (1.21 µg/mL) and the lowest contents at the 24th week of lactation (1.04 µg/mL). To the best of our knowledge, this is the first study investigating Sr and Ba milk content variation through lactation and for this reason comparison with other studies dealing with this topic is not possible.

The increase of major mineral contents in milk through lactation could be the consequence of the concentration effect due to low milk yield at the end of lactation [35]. However, the greater concentration of minerals at the end than at the beginning of lactation could also depend on extrinsic factors such as seasonal conditions (temperature and rainfall), soil and phenological state of plant as reported by Qeshlagh et al. [36] who detected greater Ca, P and Na contents in ewe milk in summer than spring grazing season. The greatest B sources are food and drinking water [32]. In this study, the B content fluctuate across lactation. The greater mineral content in late lactation could result in better milk coagulation properties than in early lactation; indeed, Malacarne et al. [31] affirmed that milk richer in Ca, P and Mg influence positively milk coagulation properties (low rennet coagulation time and curding firming time with great curd firmness) in agreement with Vacca et al. [26] who found that goat milk at the end of lactation showed better rennet coagulation time, shorter curd-firming time and a greater curd firmness compared with milk at the beginning of lactation. 

## 5. Conclusions

The results of the present study contribute to the characterisation of milk from Italian local goat breeds with regards to mineral content and compare the milk mineral profiles of local breeds and the cosmopolitan Saanen breed. Small differences among breeds were observed for major and trace minerals in milk, being significant only for P, Mg and Zn. Although SA yielded more milk with greater SCS and lower fat concentration than the local breeds, this breed had the same P, Mg and Zn milk content than local breeds. Week of lactation affected significantly all major and trace minerals in milk (except for B), and the greatest contents for almost all the minerals were observed at the end of lactation, likely due to a concentration effect. Mineral fraction in milk is important for a technological point of view as well as for human health, thus the characterisation of the mineral profile of local goat breeds is a possible strategy to valorise the autochthonous breeds and preserve the biodiversity.

## Figures and Tables

**Table 1:** Descriptive statistics of goat milk yield, composition and mineral contents.

Trait	N. of Observation	Mean	SD	Minimum	Maximum
Milk yield (kg/day)	815	1.18	0.53	0.20	3.25
FCM3.5% (kg/day)^1^	815	1.21	0.50	0.22	3.18
Fat (%)	815	3.97	1.06	1.90	8.81
Protein (%)	815	3.36	0.56	2.10	5.61
Lactose (%)	815	4.48	0.33	3.30	5.41
SCS (units)^2^	815	5.61	1.90	0.36	10.17
Major minerals (mg/kg)					
K	217	1662	231	1117	2278
Ca	217	1067	193	480	1593
P	217	796	127	462	1122
Na	217	348	71	241	650
S	217	293	50	150	411
Mg	217	107	22	64	169
Trace elements (µg/g)					
Zn	217	2.70	0.74	0.67	4.74
B	217	1.42	0.80	0.04	3.71
Sr	217	0.81	0.28	0.05	1.52
Ba	217	0.35	0.32	0.06	1.92
Fe	217	0.31	0.16	0.06	0.98

^1^ FCM3.5% = milk yield (kg/day) × (0.634 + 0.1046 × fat%); ^2^ SCS = 3 + log_2_(SCC/100 000).

**Table 2:** Least squares means of milk yield, composition and mineral contents of 6 goat breeds.

Traits	Breed^1^						Overall	
	GA	GI	JO	MA	MR	SA	SEM	*p*
Milk yield (kg/day)	0.99^d^	0.93^d^	1.25^bc^	1.28^b^	1.01^cd^	1.55^a^	0.10	***
FCM3.5% (kg/day)^2^	1.01^b^	1.00^b^	1.30^a^	1.33^a^	1.05^b^	1.50^a^	0.08	***
Fat (%)	3.90^a^	4.42^a^	4.10^a^	4.04^a^	4.13^a^	3.26^b^	0.16	***
Protein (%)	3.71^a^	3.27^bc^	3.36^bc^	3.11^c^	3.40^b^	3.42^ab^	0.08	***
Lactose (%)	4.26^b^	4.47^a^	4.53^a^	4.49^a^	4.58^a^	4.28^b^	0.04	***
SCS (units)^3^	5.90^b^	5.05^c^	5.54^bc^	5.04^c^	5.46^bc^	6.78^a^	0.27	***
Major minerals (mg/kg)								
K	1753	1753	1605	1587	1585	1667	32	
Ca	1073	975	1144	1041	1100	1093	24	
P	784^ab^	756^b^	833^a^	716^b^	882^a^	817^ab^	24	***
Na	374	332	353	340	326	359	7	
S	305	277	302	277	308	288	6	
Mg	114^ab^	102^ab^	107^ab^	95^b^	111^ab^	120^a^	4	*
Trace elements (µg/g)								
Zn	2.65^ab^	2.46^b^	3.17^a^	2.29^b^	3.19^a^	2.49^b^	0.16	***
B	1.32	1.33	1.36	1.57	1.32	1.56	0.05	
Sr	0.75	0.75	0.97	0.80	0.80	0.80	0.03	
Ba	0.25	0.35	0.42	0.24	0.36	0.40	0.03	
Fe	0.33	0.31	0.30	0.27	0.33	0.35	0.04	

^1^ GA = Garganica; GI = Girgentana; JO = Jonica; MA = Maltese; MR = Mediterranean Red; SA = Saanen; ^2^ FCM3.5% = milk yield (kg/day) × (0.634 + 0.1046 × fat%); ^3^ SCS = 3 + log2(SCC/100 000); ^abc^ Least squares means with different superscripts within a row differ significantly (*p* < 0.05); * *p* < 0.05,** *p* < 0.01, *** *p* < 0.001.
